# Computational analysis of Ayurvedic herbs to explore their potential role as anti-cervical cancer agents

**DOI:** 10.22099/mbrc.2024.51173.2038

**Published:** 2025

**Authors:** Suhani Dange, Neha Aggarwal, Rivi Verma, Yashika Sinha, Sonakshi Dadhiya, Gagan Mittal, Ruchi Sachdeva

**Affiliations:** 1 Department of Bioinformatics, Goswami Ganesh Dutta Sanatan Dharma College, Sector-32, Chandigarh, India; 2 Department of Biotechnology, Panjab University, Chandigarh, India; 3 Department of Zoology, RKSD College, Kaithal, Haryana, India

**Keywords:** Anti-cervical cancer, Ayurvedic herbs, Pathways, Protein targets, Bioactive compounds

## Abstract

Cervical cancer is one of the common types of cancer in women. Treatment regimens include use of chemotherapy but it leads to certain side effects thereby creating a need for safer therapeutic options. Ayurveda has a great potential to provide better treatment strategies. In this study, computational approaches have been employed to investigate the molecular mechanism of anti-cervical cancer Ayurvedic herbs. Initially, Ayurvedic plants possessing anti-cervical cancer activities were obtained from literature. Bioactive compounds present in such plants were evaluated for drug-likeliness, biological functions and associations with cancer-related pathways. This resulted in the most promising drug-like bioactive compounds which were found to target cancer pathways like microRNA and proteoglycans, Human papillomavirus infection. Anti-cervical cancer activity possessing herbs derived from the study include *Camellia sinensis, Equisetum arvense, Rosmarinus officinalis.* Major bioactive compounds extracted from the enlisted herbs that contribute in promoting anti-cervical cancer effects include allicin, apigenin, and mataresinol. Overall, our study has provided insights into the scientific mechanism behind anti-cervical cancer activities of the indigenous herbs of Ayurveda. In addition, this study has also highlighted key bioactive compounds which have a potential in targeting cancer related pathways and thus can further be utilized to devise better therapeutics to cure cervical cancer.

## INTRODUCTION

Across the globe, cervical cancer shows high rates of occurrence and mortality rate making it the second leading cause of death in women worldwide [1]. Cervical cancer is the world's fourth leading cause of cancer in women and the second most prevalent malignancy in women aged 15 to 44 [2]. According to the World Health Organization's cervical cancer profile of India, in 2020, the crude cervical cancer incidence and the age-standardized cervical cancer incidence per 100,000 women is 18.7 and 18, respectively. The cumulative risk of cervical cancer for people aged 0 to 74 is 2.0% and the mortality-to-incidence ratio (2020) is 0.62. These figures support the necessity for additional research in the field of cancer treatment [3]. As cervical cancer ranks fourth for both incidence and mortality, it is significantly more prevalent in low and middle-income countries [4]. In high and low-resource countries, the maximum incidence occurs around the age of 40-69 years of age with the global average age of death from cervical cancer of 59 years. With 97,000 cases and 60,000 fatalities, India contributes more than a third of the worldwide cervical cancer burden. Cervical cancer was among the top three cancers affecting women in 146 (79%) of the 185 nations studied [5].

Based on the origin of the malignancy, cervical cancer is usually divided into two types: squamous cell carcinoma and adenocarcinoma [6]. The cells that line the cervix are affected by cervical cancer, which most frequently affects the cells in the transformation zone [7]. The interference of the tumour suppressor proteins p53 and pRB (retinoblastoma) with viral oncoproteins E6 and E7 is a major factor in the development of cervical cancer [8]. Women who have been infected with the human papillomavirus are more likely to develop cervical cancer [6]. Cervix precancerous alterations normally do not produce discomfort and are not diagnosed unless a woman is screened. Symptoms do not usually occur until abnormal cervical cells are malignant and infect adjacent tissue. The most common symptoms are excessive foul-smelling vaginal discharge, atypical bleeding or inter-menstrual bleeding, post-coital haemorrhage, postmenopausal bleeding, or backache [9].

Treatment strategies include chemotherapeutic drugs which can be used to treat illnesses or invasive and pricey surgical and ablative methods. But these are not readily available by millions of patients, especially in developing nations. The availability of potent natural therapies directed against the virus is thus one of the key alternatives to treat HPV-related disorders [8]. According to the American Cancer Society, chemotherapy medications kill cancer cells while also damaging normal cells, which can result in side effects. Its short-term effects include nausea and vomiting, loss of appetite, hair loss, mouth sores, and exhaustion. It can harm the bone marrow's blood-producing cells, causing blood cell levels to fall. Chemotherapy can also cause menstruation abnormalities, neuropathy, and nephrotoxicity in the long run.

The use of plant-based medicines has gained attention during the last few years. Because of their accessibility, availability, inherited practice, economic feasibility, and perceived efficacy, medicinal plants have been utilized to cure many ailments from ancient times [10]. Since ancient times, Ayurveda has been a traditional healthcare system of Indian medicine. Several Ayurvedic medications that are used to treat and manage a variety of disorders in humans, have been produced and practiced as 'tradition to trend' from Ayurveda to current practice [11]. Plant-based pharmaceuticals with potent anticancer properties may serve as low-cost medication with few clinical adverse effects. 

In this paper, an attempt has been made to investigate the potential of anti-cervical cancer Ayurvedic herbs using modern scientific validation methodologies including bioinformatics and systems biology. Our study has not only provided the scientific rationale for the desired activity but has also screened the most promising herbs which can further be utilized for designing better therapeutic leads to cure cervical cancer.

## MATERIALS AND METHODS

### Collecting information on herbs with anti-cervical cancer properties:

Scientific literature was searched to obtain information on Ayurvedic herbs which possess anti-cervical cancer properties as indicated by experiments. The search was carried out using NCBI PubMed. Herbs were further filtered, manually studied and were referred to as anti-cervical cancer herbs (ACCH).

### Determination of bioactive compounds present in anti-cervical cancer herbs:

The anti–cervical cancer herbs derived from the previous step were further inspected to identify the bioactive compounds present in them. These compounds were identified using three databases: IMPPAT (Indian Medicinal Plants, Phytochemistry and Therapeutics) [12], Duke’s Phytochemical database (http://phytochem.nal.usda.gov) and PCIDB (Phytochemical Interactions DB). SMILES annotations of compounds were obtained from PubChem [13] and ChEMBL [14] databases. Only the compounds having SMILE annotations available were considered.

### Evaluation of drug-likeliness and ADMET parameters of Bioactive compounds:

 Assessment of ADMET (Absorption, Distribution, Metabolism, Excretion and Toxicity) properties during initial stages of drug discovery is a crucial step as it reduces the chances of pharmacokinetics related failures during later stages [15]. The ADMET parameters were assessed based on Lipinski’s rule of five which states that drug-like compounds possess properties such as molecular weight less than 500 Dalton, a number of H-bond acceptors less than 10, a number of H-bond donors less than 5 and log P value of less than 5. Other criteria such as Caco-2 permeability (permeability coefficient that predicts the absorption of orally administered drugs) and toxicity were used for screening the drug-like bioactive compounds with the help of pkCSM [16, 17]. The cut-off threshold values of Caco-2 permeability score was taken as 0.9. In addition, negative results for two types of toxicity parameters- Hepatotoxicity and AMES toxicity were also considered. The bioactive compounds satisfying the above criterion were shortlisted and considered for further analysis.

### Classification of drug-like bioactive compounds (DBACs):

Classification of the DBACs was done by using the ClassyFire tool [18]. This tool provides annotation of chemical compounds using chemical taxonomy and structure-based ontologies. The detailed information about the broad and detailed chemical classes of the DBACs was obtained.

### Identification of protein targets of DBACs:

In this step, the potential protein targets of DBACs were predicted using three databases. Interaction pairs with high confidence scores of ≥0.85 were searched using BindingDB that contains the protein interaction information of the potential drug targets with their respective ligands [19]. Another database used was STITCH 5.0 which catalogues the information of both manual as well as experimentally curated protein-chemical interactions [20]. The confidence score of ≥0.7 was considered for searching. SwissTargetPrediction [21] was also used where search was limited to “Homo sapiens” and top 15 protein targets were predicted.

### Analysis of biological pathways:

The protein targets were further inspected for their role in various biological pathways with the help of KEGG (Kyoto Encyclopaedia of Genes and Genomes) database [22]. In addition, proteins known to be involved in different human cancer pathways were extracted from two databases KEGG [22] and Reactome [23].

### Network construction and analysis:

The obtained datasets on anti-cervical cancer herbs, their DBACs and protein targets were used for construction of bipartite networks. Cytoscape (version 3.0) [24] was used for the network construction, visualization and analysis of three networks: (1) Herb-Bioactives (H-B) network; (2) Drug-like Bioactive compounds-Protein target (DB-PT) network and (3) Protein Target-Human pathway (PT-HP) network (protein targets and their human pathways).

### Screening of potential anti-cervical cancer bioactive compounds:

For analysing the anti-cancer potential of DBACs, the following selection criteria were used. The DBACs and protein targets satisfying the given conditions were included. 

### Preparation of ‘cancer proteins dataset’:

a) Firstly, the data of the DBACs predicted from at least 2 out of the 3 databases used (BindingDB, STITCH, SwissTargetPrediction) was selected. The protein targets of these filtered DBACs were considered. b) Next, the protein targets identified from the first criteria were further confirmed with the “Approved list of protein targets” from DrugBank [25]. Only those targets present in the approved list were finally selected. c) Amongst the selected protein targets, those involved in at least 5 human pathways associated with cancer were finally considered as ‘cancer proteins dataset’. 

### Selection criteria:

 For screening the most promising anti-cervical cancer bioactive compounds, DBACs which were found to fulfil any one of the two selection criteria were shortlisted. 

Selection criteria 1. DBACs which target at least 5 proteins belonging to the ‘cancer proteins dataset’. 

Selection criteria 2. DBACs which target at least two proteins which are directly involved in cervical cancer. 

## RESULTS

In the beginning of the study, we identified 46 anti-cervical cancer herbs from literature. A unique ACCH identifier was assigned to each herb and its detailed information including herb ID, scientific name and common name were collected. The collected data on anti-cervical cancer herbs along with their references is listed in supplementary Table S1. A number of bioactive compounds present in the herbs were derived using different databases as mentioned in the methodology. The information of these bioactive compounds along with their SMILES annotation were obtained (Supplementary Table S2). These bioactive compounds were assigned a unique ACCB identifier. This dataset contains 604 bioactive compounds with 481 unique entries.

**Figure 1:**
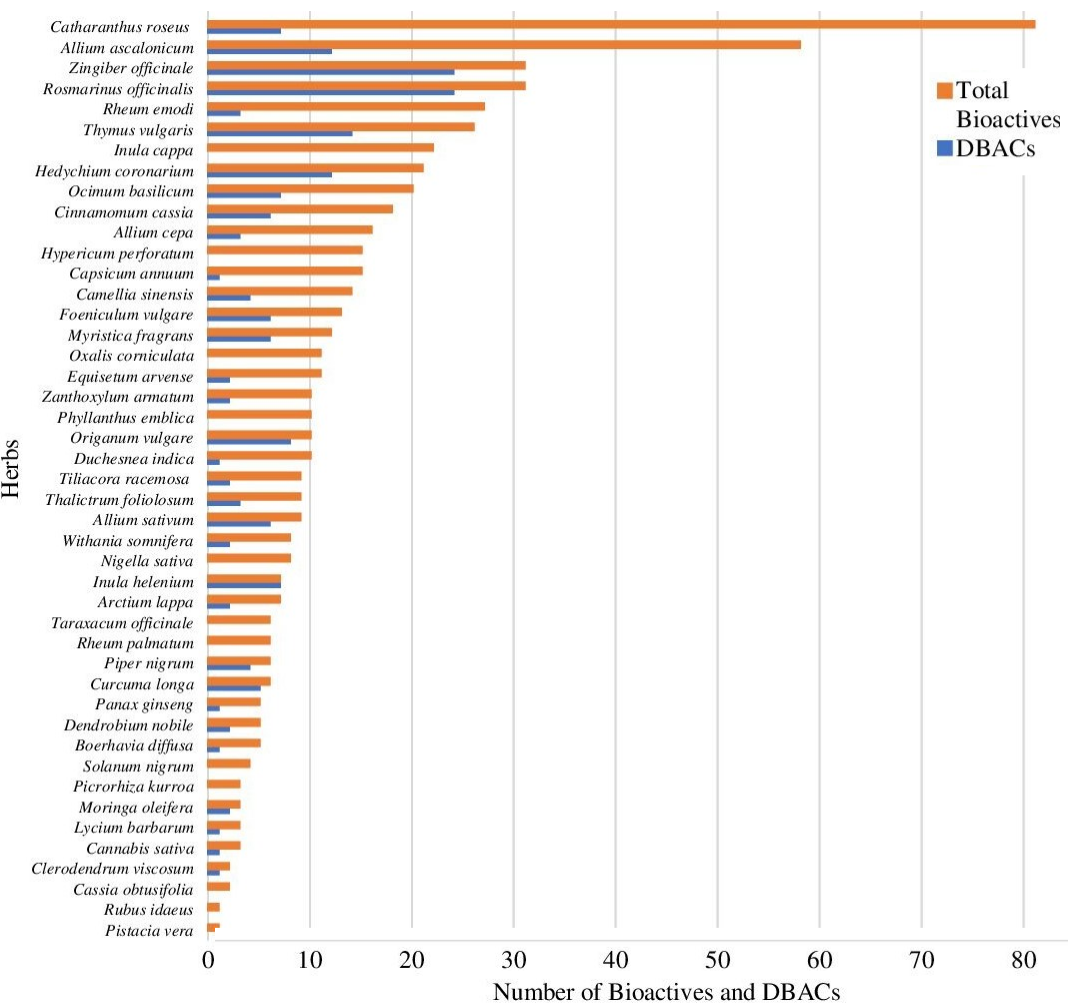
Number of bioactive and drug-like bioactive compounds present in anti-cervical cancer herbs

Out of 450 bioactive compounds, 134 were found to have drug-like properties as predicted by a variety of tools. Details of pkCSM prediction results are provided in supplementary Table S3. SMILES notations along with PubChem IDs of drug-like bioactive compounds (DBACs) are listed in supplementary Table S4. Among these DBACs, the compounds beta-pinene (ACCB135), linalool (ACCB350) and alpha-phellandrene (ACCB100) are shared by 7, 7 and 6 ACC herbs, respectively. ACC herbs such as *Zingiber officinale* (ACCH47) and *Rosmarinus officinalis* (ACCH37) were found to have a maximum number of DBACs i.e. 24 each (Fig. 1). 

**Figure 2:**
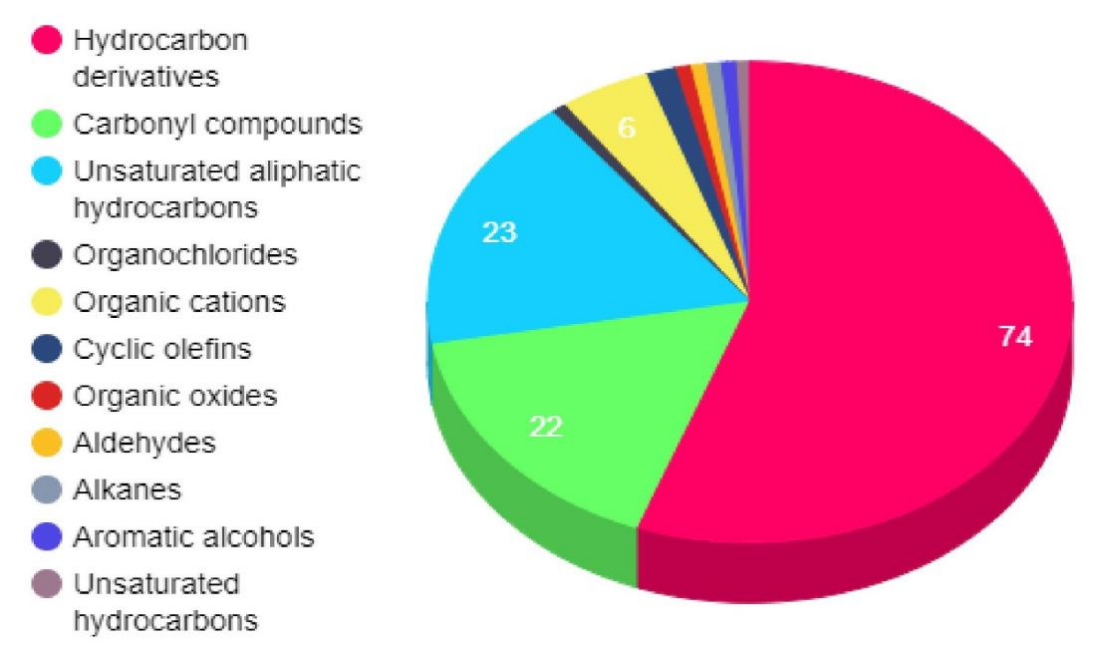
Chemical classification of drug-like bioactive compounds

The comprehensive organization of 134 drug-like bioactive compounds (DBACs) was found to be distributed in the 11 broad chemical classes. Detailed classification of the DBACs reveals that the chemical class corresponding to terpenoids, especially “sesquiterpenoids” is highly prevalent in this dataset (Fig. 2). In addition, from the same class of terpenoids, compounds belonging to monoterpinoidsincluding bicyclic monoterpinoides, menthanemono-terpinoids and acyclic monoterpinoids are also significantly high in number. Other than the class of terpenoids, compounds belonging to the detailed chemical class “Eudesmanolides, secoeudesmanolides and their derivatives” were also comparatively high in number. 

**Figure 3:**
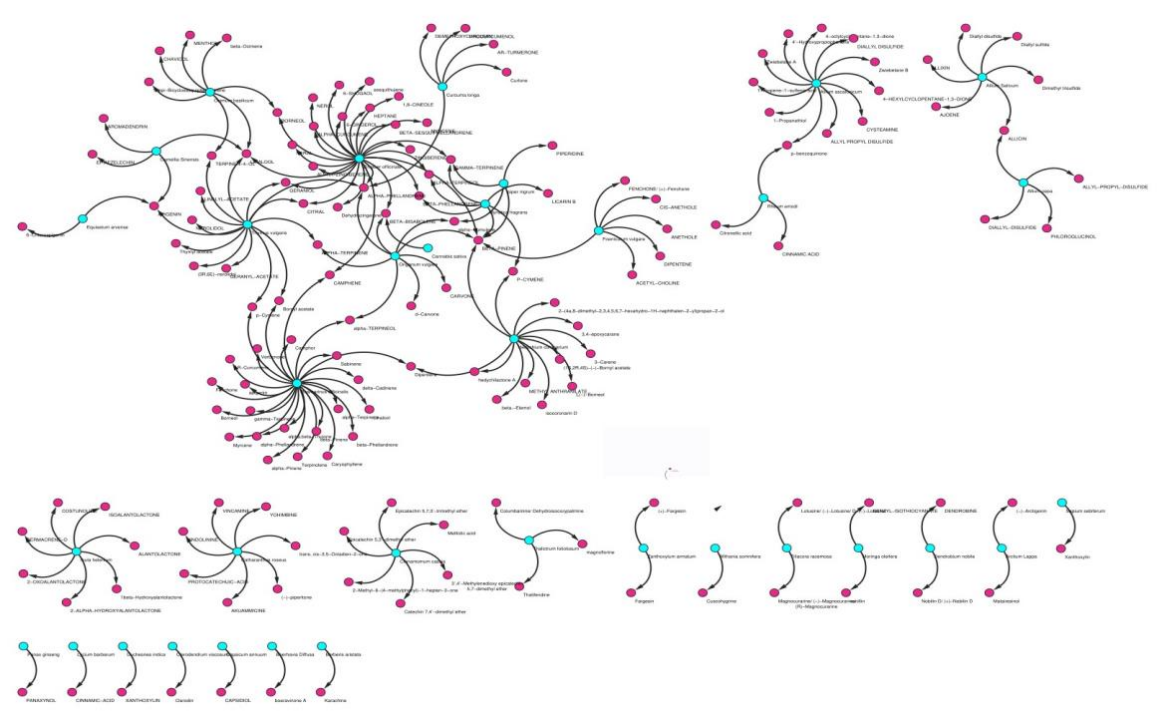
Herb-bioactive network (H-B). The figure shows the interaction of anti-cervical cancer herbs with their bioactive compounds in the form of a network involving edges and nodes. It depicts herbs as blue nodes and bioactives as pink nodes. The edges in arrow style show the direction of relation.

The information on herbs and their bioactive compounds was used to construct Herb-Bioactives (H-B) interaction networks (Fig. 3). Although several bioactive compounds are shared by the set of anti-cervical cancer herbs, some bioactives are specific in nature. ACCB375 (phytosterols) is the most common bioactive compound as it is found in 8 out of 47 ACC herbs. Other bioactives enriched in the ACCHs are ACCB350 (linalool), ACCB135 (beta-pinene), ACCB386 (quercetin) and ACCB252 (eugenol) occurring in 7, 7, 6 and 6 herbs, respectively. Some bioactives were found to be unique to specific herbs. Few of these include ACCB002 ((-)-anaferine) was found in *Tiliacora racemosa*, ACCB004 ((-)-arctigenin) in *Arctium lappa*, ACCB007 ((-)-piperitone) in *Catharanthus roseus*, ACCB066 (6-Shogaol) in *Zingiber officinale, *ACCB157 (Capsidiol) in *Capsicum annuum *and ACCB302 (Karachine) in *Berberis aristata.*

**Figure 4:**
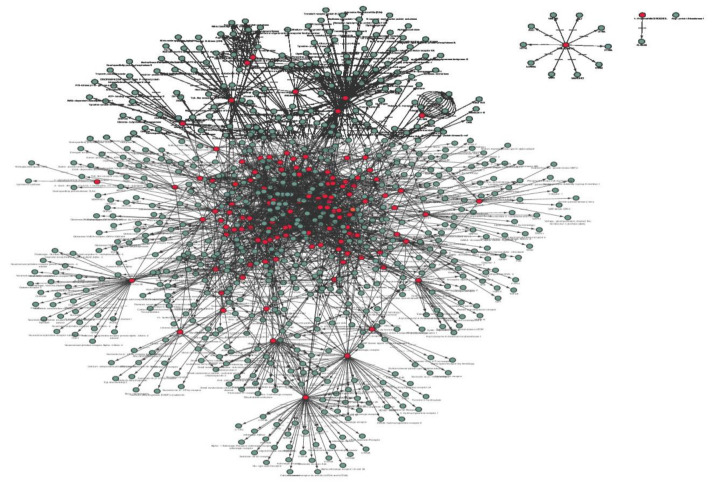
Drug-like bioactive compound-Protein target network (DB-PT). The figure shows the interaction of DBACs with protein targets in the form of a network involving edges and nodes. It depicts DBACs as red nodes and their protein targets as green nodes.

DBACs were further investigated for their potential protein targets. 134 DBACs were found to have a total of 2239 protein targets (PTs). Out of these 2239 protein targets, unique entries were 647. To understand the molecular interactions of DBACs with human proteins, a bipartite DB-PT network was constructed (Fig. 4). DBACs such as apigenin (ACCB111), yohimbine (ACCB476), 6-chloroapigenin (ACCB060) and magnoflorine (ACCB326) possess a large number of protein targets i.e. 83, 61, 48 and 48, respectively. It was revealed from the DB-PT network that the protein targets cytochrome P450 1A1, acetylcholinesterase and androgen receptor were found to be maximally associated with DBACs.

**Figure 5:**
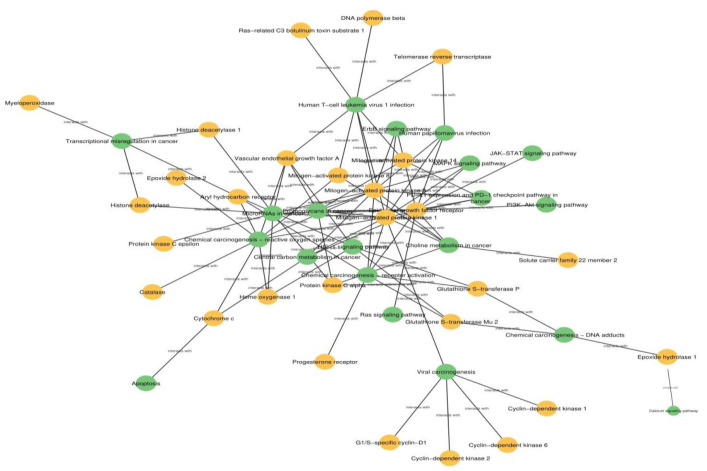
Protein Target-Human Pathway network (PT-HP). The network comprises protein targets as yellow nodes and human pathways (shown as green nodes) targeted by them.

Functional analysis yielded a number of pathways where the protein targets play a crucial role (see supplementary Table S5 for details). Only 130 protein targets showed their involvement in the human pathways as predicted by KEGG database. PT-HP network illustrates the association of protein targets (protein targets of DBACs) with human pathways based (Fig. 5). It was observed from the network that out of the given set of protein targets, six protein targets (cathepsin K, cathepsin S, cathepsin B, cytochrome c, NADPH oxidase 4 and protein kinase C alpha) were involved in >=10 human pathways. Notably, the protein targets such as epidermal growth factor receptor (EGFR), mitogen-activated protein kinase 1 (MAPK1) and mitogen-activated protein kinase 3 (MAPK3) were found to maximally participate in >10 cancer pathways. The cancer pathways enriched with the protein targets were ‘chemical carcinogenesis-reactive oxygen species’ and ‘microRNAs in cancer’.

Based on the criteria mentioned above in Section 2.8.1 of methodology, 14 potential protein targets were obtained for the ‘cancer proteins dataset’. These include caspase-3, cellular tumour antigen p53, cyclin-dependent kinase 6, dual specificity mitogen-activated protein kinase 1, epidermal growth factor receptor, vascular endothelial growth factor A, hypoxia-inducible factor 1-alpha, mitogen-activated protein kinase1, mitogen activated protein kinase 3, RAC-alpha serine/threonine-protein kinase, receptor-type tyrosine- protein kinase FLT3, serine/ threonine protein kinase B-raf, serine/threonine-protein kinase mTOR, tyrosine-protein kinase JAK2 and tyrosine-protein kinase SRC. 

**Figure 6:**
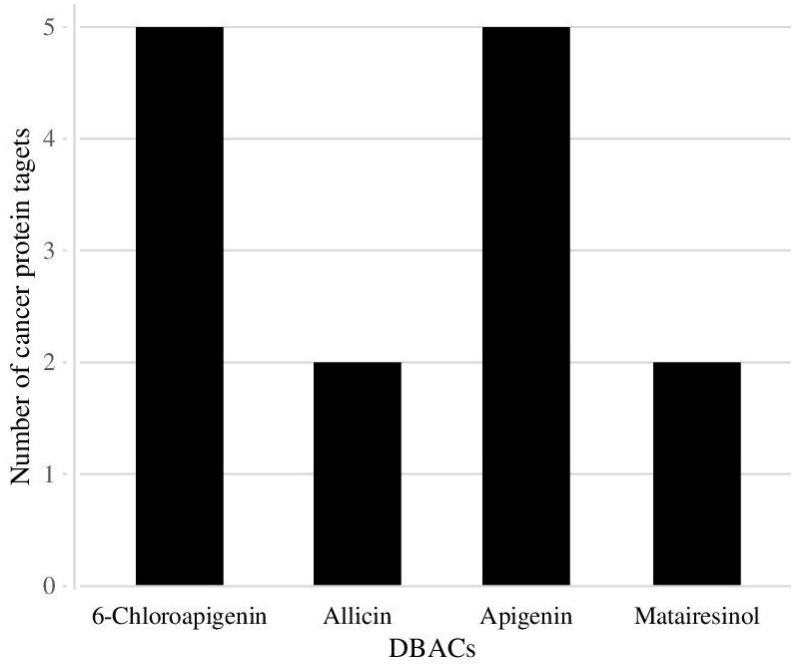
The graph highlights the most promising DBACs which target the maximum number of cancer specific proteins.

According to the Selection criteria 1 (Section 2.8.2), two DBACs were found to target at least 5 protein targets included in the ‘cancer proteins dataset’ (Table 1). By applying selection criteria 2, four DBACs were observed to target at least two proteins directly involved in cervical cancer (Table 2). Among these, common DBACs satisfying both the criteria were apigenin and 6-chloroapigenin. Collectively, the most promising DBACS found were apigenin, 6-chloroapigenin, allicin and matairesinol which have the potential of targeting a maximum number of cancer specific proteins (Fig. 6).

In broad chemical classification, apigenin and allicin belong to the chemical class of hydrocarbon derivatives. The compounds 6-Chloroapigenin and matairesinol belong to the broad chemical class of “organochlorides” and “carbonyl compounds”, respectively. In detailed chemical classification, both apigenin and 6-chloroapigenin belong to the chemical class of “flavones”. Allicin belongs to the detailed chemical class of “thiosulfinic acid esters” and matairesinol belongs to “dibenzylbutyrolactone lignans”.

**Figure 7:**
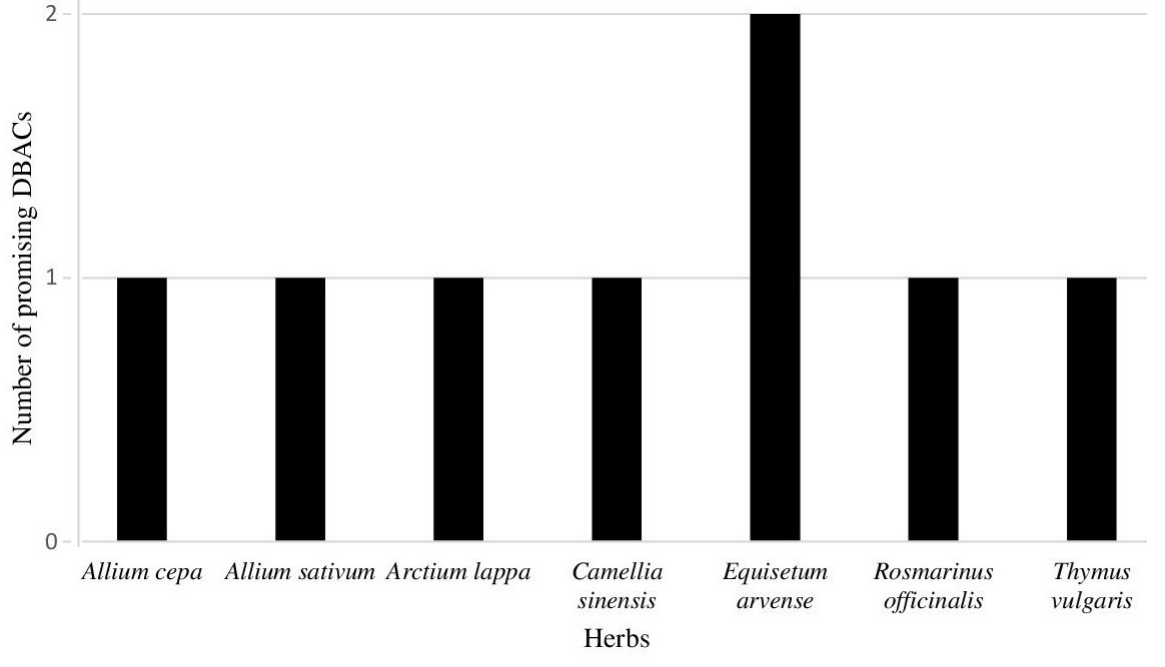
The graph shows herbs which contain the most promising DBACs found in our analysis.

It was observed that these promising DBACs were found in a number of herbs namely *Allium cepa, Allium sativum, Camellia sinensis, Equisetum arvense, Rosmarinus officinalis*, *Thymus vulgaris *and* Arctium lappa *(Fig. 7)*. *Also these DBACs are shown to be involved in cancer specific pathways such as such as chemical carcinogenesis - reactive oxygen species, choline metabolism in cancer, central carbon metabolism in cancer, calcium signalling pathway, chemical carcinogenesis - receptor activation, MAPK signalling pathway, Ras signalling pathway. Among these, pathways such as human papillomavirus infection, viral carcinogenesis, cell cycle, PI3K-Akt signalling pathway, loss of function of TP53 are known to be important in cervical cancer.

## DISCUSSION

Our study has found that “sesquiterpenoids” are highly prevalent bioactive compounds present in ACC herbs. These compounds are a class of 15-carbon isoprenoid molecules that possess therapeutic potential in reducing the spread of cancer [26]. Other prominent bioactive compounds found in ACC herbs, such as monoterpenoids geraniol, nerol, geranial and neral compounds have been proven to be beneficial for multifactorial complex diseases like cancer, in reducing tumour cell resistance [27]. Previous studies have also reported that these compounds show antineoplastic activity in animal and cell models in numerous cancer types and activate multiple antitumor responses such as apoptosis and necrosis [27]. 

Our study has highlighted the most promising bioactive compounds which were found to be associated with cancer related targets and biological pathways. One such compound is apigenin, a plant-derived flavonoid that has been reported as an anticancer agent in several experimental studies. It has been shown to exhibit cell growth arrest and apoptosis in different types of tumours such as breast, lung, prostate, pancreatic, cervical, oral, and stomach, by modulating several signalling pathways [28]. According to a study, apigenin has been demonstrated to affect HeLa cell autophagy and death by controlling the PI3K/Akt/mTOR signalling pathway [29].

Another significant DBAC is allicin (diallyl thiosulfinate) found in garlic. It has great inhibitory effects at high doses and significantly inhibits the ability of Hela and Siha cells to proliferate, invade, and migrate. Among the many benefits of allicin are its anticancer, antibacterial, anti-free radical, anti-inflammatory, and immune system-regulating properties. Additionally, a study has reported a certain inhibitory effect of allicin on the invasion and metastasis of various tumor cells, including liver cancer cells [30].

Matairesinol is a type of lignan which is found abundantly in fruits, vegetables, and seeds of plants. Previous research has reported pharmacological and biological characteristics of matairesinol, along with its anti-cancer property in a variety of cancer types, including pancreatic, breast, cervical and prostate cancer [31]. At non-toxic concentrations, it has been shown to reduce hypoxia-inducible factor-1a in hypoxic HeLa cells [32].

Our study has indicated that *Thymus vulgaris *contains numerous bioactive compounds which have the most potential anticancer activities*. *Previous study has suggested that *Thymus vulgaris* extract reduces the proliferative, migratory, and invasive abilities of HCT116 cells, a human colon cancer cell line [33]. The human colon cancer cell line HCT 116 is frequently employed in research on the biology of cancer. Research conducted in vitro has demonstrated that this growth factor-independent cell line is highly motile and invasive [34]. Although its efficacy has been proven for colon cancer and colorectal cancer, our study has highlighted its potential for treatment for cervical cancer. 

Rosemary (*Rosmarinus officinalis L.*) is a commonly used culinary and medicinal herb. A study has shown that rosemary extract significantly inhibits the growth of human ovarian cancer cells by interfering with the cell cycle. It has the potential to be used in conjunction with chemotherapy for cancer since it increases apoptosis by altering the expression of numerous genes that control apoptosis [35]. Our study has indicated an additional role of *Rosemary* as a therapeutic alternative for curing cervical cancer.

*Allium sativum L. *is an invaluable herb as it possesses numerous health benefits such as antithrombotic, antimicrobial, hypoglycemic, antitumor and hypolipidemic properties. Its anticancer properties are mostly attributed to its sulphur-containing components, which also affect different drug-metabolizing enzymes, free radical scavenging activity and anti-oncogenesis. Additionally, it contains phytochemicals that have the potential to prevent cancer by modulating cell signalling pathways, arresting unwanted cell proliferation and modulating allyl isothiocyanate [36].

*Allium cepa* displays a rich phytochemistry as it contains a combination of fructans, flavonoids, and organosulfur compounds. Previous research has demonstrated that the naturally occurring components of onion possess antitumor properties and cause oxidative stress-induced apoptosis [36]. Furthermore, a study on diallyl trisulfide (DATS) found in *Allium* vegetables like onion and garlic, has shown that DATS causes cancer cell cycle arrest at the G2/M phase by increasing the release of reactive oxygen species, which in turn triggers apoptosis and limits the formation of tumour cells [37].

*Arctium lappa *has been shown to hinder the growth of tumours and may be useful in the treatment of malignant melanoma, ovarian, bladder and breast tumours [38]. Several lines of evidence have indicated that *Arctium lappa* significantly inhibits cell migration and invasion in human cervical cancer cells mainly by reducing phosphoinositide 3-kinase (PI3K) and phosphorylation of Akt [39].

*Camellia sinensis* (green tea) has been shown to possess antiviral and immunomodulatory properties which may confer chemoprotective effects thereby providing protection against cancers linked to HPV. In addition, a study has shown that green tea polyphenols cause dose-dependent apoptosis which prevents the proliferation of HPV16 cervical cancer cells [40]. *Equisetum arvense* has also been reported to possess significant cytotoxic activity against various types of cancer cell lines and normal cell lines in vitro [41]. 

Our study has highlighted a significant role of the MAPK pathway which is being targeted by the most promising DBACS. MAPK pathway functions to regulate numerous processes such as apoptosis, migration, differentiation, proliferation, and growth. A study has reported that expression of HPV oncoproteins like E6 and E7 increases the activity of the different effectors for MAPK pathways and suppresses the p53 protein which leads to the growth of cancerous tumours and apoptosis prevention. In many cervical cancer cases, ERK/MAPK has been found to be involved in the activation and overexpression of oncogenes derived from the cells. EGFR and VEGF have been reported to be the two important signalling pathways involved in the proliferation of HPV-related cancers [42].

Another critical pathway found was the ErbB signalling pathway of ErbB that belongs to the epidermal growth factor receptor family. A study has shown that blocking ErbB2 leads to apoptosis of cervical cancer cells [43]. Another research has shown that HOXC cluster antisense RNA 3 (HOXC-AS3) and up-regulating Sevenless Homolog 1 (SOS1) increases cervical cancer cell proliferation, migration and invasion by enhancement of the ErbB signalling pathway [44].

To conclude, our study has not only provided the scientific rationale behind the anti-cervical cancer properties of Ayurvedic herbs but also screened the most promising herbs and drug-like bioactive compounds which can further be employed as lead compounds for drug development.

**Table 1:** DBACs targeting at least 5 protein targets included in ‘cancer proteins dataset’ [Selection criteria 1].

**S. No.**	**DBACs**	**Herbs**	**Protein targets included in ‘cancer proteins dataset’**
1	Apigenin	*Camellia sinensis* *Equisetum arvense* *Rosmarinus officinalis* *Thymus vulgaris*	Caspase-3 Cellular tumour antigen p53 Epidermal growth factor receptor RAC-alpha serine/threonine-protein kinase Receptor-type tyrosine-protein kinase FLT3
2	6-Chloroapigenin	*Equisetum arvense*	Caspase-3 Cellular tumour antigen p53 Epidermal growth factor receptor RAC-alpha serine/threonine-protein kinase Receptor-type tyrosine-protein kinase FLT3

**Table 2:** DBACs targeting at least two proteins directly involved in cervical cancer [Selection criteria 2].

**S. No.**	**DBACs**	**Herbs**	**Protein targets directly involved in cervical cancer**
1	Apigenin	*Camellia Sinensis* *Equisetum arvense* *Rosmarinus officinalis* *Thymus vulgaris*	Cellular tumour antigen p53 Epidermal growth factor receptor RAC-alpha serine/threonine-protein kinase
2	6-Chloroapigenin	*Equisetum arvense*	Cellular tumour antigen p53 Epidermal growth factor receptor RAC-alpha serine/threonine-protein kinase
3	Allicin	*Allium cepa* *Allium Sativum*	Mitogen-activated protein kinase 1 Mitogen-activated protein kinase 3
4	Matairesinol	*Arctium lappa*	Dual specificity mitogen-activated protein kinase kinase 1 Serine/threonine-protein kinase B-raf
